# The Transcriptional Response of *Caenorhabditis elegans* to Ivermectin Exposure Identifies Novel Genes Involved in the Response to Reduced Food Intake

**DOI:** 10.1371/journal.pone.0031367

**Published:** 2012-02-14

**Authors:** Steven T. Laing, Al Ivens, Victoria Butler, Sai P. Ravikumar, Roz Laing, Debra J. Woods, John S. Gilleard

**Affiliations:** 1 Faculty of Veterinary Medicine, University of Glasgow, Glasgow, Strathclyde, United Kingdom; 2 Fios Genomics Ltd, The Edinburgh Technology Transfer Centre, Edinburgh, Lothian, United Kingdom; 3 Faculty of Veterinary Medicine, University of Calgary, Calgary, Alberta, Canada; 4 Research and Development, Pfizer Animal Health, Kalamazoo, Michigan, United States of America; New England Biolabs, United States of America

## Abstract

We have examined the transcriptional response of *Caenorhabditis elegans* following exposure to the anthelmintic drug ivermectin (IVM) using whole genome microarrays and real-time QPCR. Our original aim was to identify candidate molecules involved in IVM metabolism and/or excretion. For this reason the IVM tolerant strain, DA1316, was used to minimise transcriptomic changes related to the phenotype of drug exposure. However, unlike equivalent work with benzimidazole drugs, very few of the induced genes were members of xenobiotic metabolising enzyme families. Instead, the transcriptional response was dominated by genes associated with fat mobilization and fatty acid metabolism including catalase, esterase, and fatty acid CoA synthetase genes. This is consistent with the reduction in pharyngeal pumping, and consequential reduction in food intake, upon exposure of DA1316 worms to IVM. Genes with the highest fold change in response to IVM exposure, *cyp-37B1*, *mtl-1* and *scl-2*, were comparably up-regulated in response to short–term food withdrawal (4 hr) independent of IVM exposure, and GFP reporter constructs confirm their expression in tissues associated with fat storage (intestine and hypodermis). These experiments have serendipitously identified novel genes involved in an early response of *C. elegans* to reduced food intake and may provide insight into similar processes in higher organisms.

## Introduction

The macrocyclic lactone ivermectin (IVM) is one of the most important drugs used for the control of animal and human parasites [1], [2]. It has been the mainstay of livestock parasite control since the early 1980s, but intensive use has led to the widespread development of drug resistance [2]–[5]. Over the last decade IVM has been increasingly used in community-wide treatment programs, aimed at the eradication of a number of human filarial parasite species. Resistance appears now to be emerging as a consequence of this intensive drug selection pressure [6]–[8]. In order to maintain IVM efficacy in regions where resistance has not arisen, and to aid in the development of novel synergists, it is essential that the mechanism of action of the drug and the molecular mechanisms employed by parasites that result in resistance are elucidated.

Genetic, molecular and electrophysiological studies on the free-living nematode *Caenorhabditis elegans* have been central to identifying the major direct molecular targets of IVM [9]–[13]. It acts by irreversibly binding to, and activating, ligand-gated ion channels, particularly glutamate-gated chloride channels (GluCls), resulting in paralysis of the body wall and pharyngeal muscles [14]–[16]. This in turn leads to generalized paralysis and decreased feeding. A number of comparative studies suggest that IVM acts in a similar way in parasitic nematodes [15]–[17].

The ultimate effect of a drug on an organism is a balance between the immediate effects of drug-receptor interaction; secondary and compensatory responses to these interactions (pharmacodynamics); and the effect of the organism on the drug (pharmacokinetics). As discussed above, the immediate effect of IVM-receptor interaction has been studied intensively, and is known to cause pharyngeal and body wall paralysis. However, the biological response of the nematode to this phenotype, and potential compensatory mechanisms induced following drug exposure, have received little attention. We have previously shown that exposure of *C. elegans* to the benzimidazole drug albendazole induces an array of xenobiotic metabolizing enzymes [18]. The transcriptomic response of nematodes to IVM exposure, a highly lipophilic drug that undergoes minimal metabolism in mammals, has not previously been described [19].

Wild-type *C. elegans* exposed to even low doses of ivermectin are rapidly paralysed leading to death. In order to minimise non-specific, stress-related changes in the transcriptome following drug exposure, we made use of the strain DA1316, which has a null mutation in three subunits of the glutamate-gated chloride channel target of IVM. Although this strain is largely resistant to the paralytic effects of IVM on the body wall, we found that following four hours exposure to high doses of the drug there is a significant decrease in pharyngeal function. As a result many of the differences in gene expression noted between the IVM exposed and unexposed groups are due to chemically induced reduced food intake of the exposed nematodes.

As well as being accepted as a model for many features of parasitic nematode biology, *C. elegans* is increasingly used as a model for the basic biology of satiety and obesity [20], [21]. There have been many studies investigating the transcriptomic response of *C. elegans* to starvation, in particular that of the long-lived, anorexic dauer stage [22], [23]. However, thus far investigation of short-term food deprivation has been limited to real-time PCR based studies of genes expected to be involved in this response [24]. To the authors' knowledge this is the first unbiased, whole-genome investigation of the immediate effects of food deprivation in *C. elegans* and in any whole organism.

## Materials and Methods

### *C. elegans* strains and maintenance

Strain DA1316 (*avr-14(ad1302*); *avr-15(vu227)*; *glc-1(pk54)*) was used in all experiments. This strain is a triple mutant of the glutamate-gated chloride channel subunits *avr-14, avr-15* and *glc-1*, conferring high-level resistance to IVM (*pers. Comm.*, Dr. J. Dent). The wild-type strain used in pharyngeal pumping assays was the Bristol N2 strain. Both strains were gifts from the Caenorhabditis Genetics Center (CGC).

### Synchronisation of cultures

Embryos were isolated by hypochlorite treatment of gravid adults [25]. The embryos were transferred to a 5 cm diameter Petri dish in 6 ml of S-buffer (129 ml/L 0.05 M K_2_HPO_4_, 871 ml/L 0.05 M KH_2_PO_4_, 0.1 M NaCl; pH 6.0), and maintained at 20°C overnight. The concentration of L1 larvae was calculated the following day and experimental cultures initiated immediately.

### Pharyngeal pumping assay

IVM (Sigma, Gillingham, Dorset, UK) plates were prepared to final concentrations of 0, 1, 10, 100 and 1000 ng/ml (0, 1.1 nM, 11.4 nM, 0.1 µM, 1.1 µM) IVM. Dimethyl sulphoxide (DMSO) was used to dissolve the IVM and was present in all plates at a final concentration of 0.01% v/v. Synchronised N2 and DA1316 L1 larvae were allowed to grow on standard nematode growth medium (NGM) plates at 20°C for 53 hr. The L4/young adults were then picked on to drug plates and allowed to remain at 20°C for a further 4 hr. The number of pharyngeal pumps was counted over a period of 1 min for five worms of each strain at each concentration of drug.

### Anthelmintic exposures

Ten thousand DA1316 L1 larvae per experimental condition were grown for 53 hr at 20°C on standard NGM plates with OP50 bacterial lawns. The nematodes were assessed for comparable staging between groups then washed from the plates with M9 buffer into a 50 ml falcon tube and washed twice in M9 buffer. The suspension of worms was split equally between control plates (DMSO 0.001% and 0.01% v/v for 100 ng/ml (0.1 µM) and 1 µg/ml (1.1 µM) IVM experiments respectively) and plates containing 100 ng/ml (0.1 µM) or 1 µg/ml (1.1 µM) IVM (Sigma, Gillingham, Dorset, UK) at a density of 500–600 worms per 5 cm diameter plate. Drug and control plates were made and seeded with 100 µl OP50 suspension 16–24 hr prior to the introduction of the larvae. After 4 hr exposure the nematodes were washed from the drug plates with M9 buffer, washed twice in M9 and the pellet of worms snap frozen and stored in liquid nitrogen until RNA extraction.

### Real-time QPCR biological replicates

Separate biological replicates were carried out for analysis by real-time QPCR (RTPCR) in an identical manner to the microarray experiments except for the use of a commercial preparation of IVM (Virbamec 5 mg/ml IVM, Virbac, Bury St. Edmunds, Suffolk, UK). In order to compare the transcriptomic response to IVM exposure and short-term food deprivation, nematodes were first grown as per the acute drug exposure protocol and then transferred either to plates containing IVM, control plates or control plates with no OP50 food source for 4 hr before harvesting. Investigation of gene up-regulation following exposure to a gradient of IVM concentrations was undertaken. Five matched cultures of *C. elegans* were grown in standard liquid culture medium for 70 hr at 20°C, 200 rpm. Cultures were exposed to 0, 1, 10, 100, 1000 ng/ml (0, 1.1 nM, 11.4 nM, 0.1 µM, 1.1 µM) IVM for 4 hr, harvested by sucrose flotation and snap frozen in liquid nitrogen [26].

### RNA methods

RNA extractions were carried out using Trizol Reagent (Invitrogen, Paisley, UK), according to the manufacturer's instructions. Harvested *C. elegans* were homogenised in four volumes Trizol reagent, subject to two chloroform extractions and precipitated in isopropanol. The RNA was then treated with RNase-free DNase I (Qiagen, Crawley, West Sussex, UK) in solution before purification and concentration using RNeasy columns (Qiagen, Crawley, West Sussex, UK). Quality and concentration of RNA was assessed using an Agilent Bioanalyser 2100. Total RNA destined for microarray analysis was re-precipitated in ethanol. RNA for real-time QPCR analysis was reverse transcribed using a cloned AMV first strand synthesis kit (Invitrogen, Paisley, UK) and random hexamer primers. 5 µg total RNA for each sample was used as template and an identical reaction lacking reverse transcriptase enzyme was carried out simultaneously as a negative control. cDNA was purified using PCR purification columns (Qiagen, Crawley, West Sussex, UK), resuspended in 30 µl TE buffer and stored at −80°C until use.

### Microarray hybridisation and analysis

Sample labelling and hybridisation to *C. elegans* whole genome Genechips (Affymetrix, High Wycombe, UK) were performed using standard Affymetrix protocols (http://media.affymetrix.com/support/downloads/manuals/expression_analysis_technical_manual.pdf). These chips contain oligonucleotide probesets designed to assess over 22500 transcripts from the *C. elegans* genome. An updated annotation dataset was assembled for the *C. elegans* probesets present on the Genechip. Data were sourced from WormBase (Sept. 2008). Scanned array images (CEL files) were quality control assessed using the arrayQualityMetrics Bioconductor package (www.bioconductor.org) in the R environment (www.r-project.org). Arrays identified as possible outliers were removed from subsequent analyses. Linear model fitting of the array data was undertaken, taking into account biological replicates using the limma (Linear Models for Microarray Data) Bioconductor package (www.bioconductor.org/packages/bioc/html/limma.html). The Rank Products algorithm was used to assess differential expression of genes between test and control groups and to assign significance to these changes [27]. Assignment of significance was carried out using a False Discovery Rate (FDR) cut-off of 5–10%.

### Gene Ontology analysis

DAVID software (the Database for Annotation, Visualisation and Integrated Discovery) from the National Institutes of Health was used to assess the functional annotation and clustering of the differentially expressed genes [28], [29]. Input into the program consisted of probesets shown to be significantly altered in expression using the Rank Products algorithm, with a false discovery rate (FDR) of less than 10%. Prevalence of annotation terms within the list of differentially expressed genes was compared to the prevalence in the whole *C. elegans* genome. Fold enrichment was calculated and a modified Fishers exact test (EASE score) used to assign significance. Gene functional classification clustering was carried out using medium stringency (DAVID).

### Real-time Quantitative PCR

Relative quantitation of genes of interest was assessed using Brilliant SYBR Green QPCR master mix (Agilent [Stratagene], Stockport, Cheshire, UK) and a Stratagene Mx 300P QPCR system with Stratagene MxPro software. *ama-1*, encoding a subunit of RNA polymerase II, was used as a normalising gene. This constitutively expressed gene showed no significant changes on microarray analysis and has been extensively used as a normalising gene in differential expression studies in *C. elegans* [30]. Gene specific primers were designed to produce a product between 160 and 200 bp in length. The sequences of these primers can be found in **Table S1**. The final concentration of primers was between 300 nM and 400 nM in a total reaction volume of 25 µl.

### Expression pattern analysis

GFP reporter constructs for *mtl-1, scl-2*, C23G10.11, *cyp-37B1*, and *ilys-3* were created using a PCR fusion protocol [31]. The putative promoter region, 3 Kb upstream from the ATG start site of the gene of interest, was fused to the *gfp* gene, including synthetic introns and *unc-54* 3′ UTR, from Fire vector pPD95.67 [32]. The plasmid used contained a *gfp* gene with a nuclear localisation signal. Primer sequences can be found in **Table S1**. Transgenic lines were created using the method of Mello *et al.*, with the plasmid pRF-4 as a co-transformation marker to identify transgenic worms [33]. Expression patterns were visualised using a Zeiss, Axioscop 2 plus microscope. Images were collected and processed using Improvision Openlab software (www.improvision.com).

## Results

### Pharyngeal pumping rate of DA1316 is reduced following exposure to IVM

**Figure 1:**
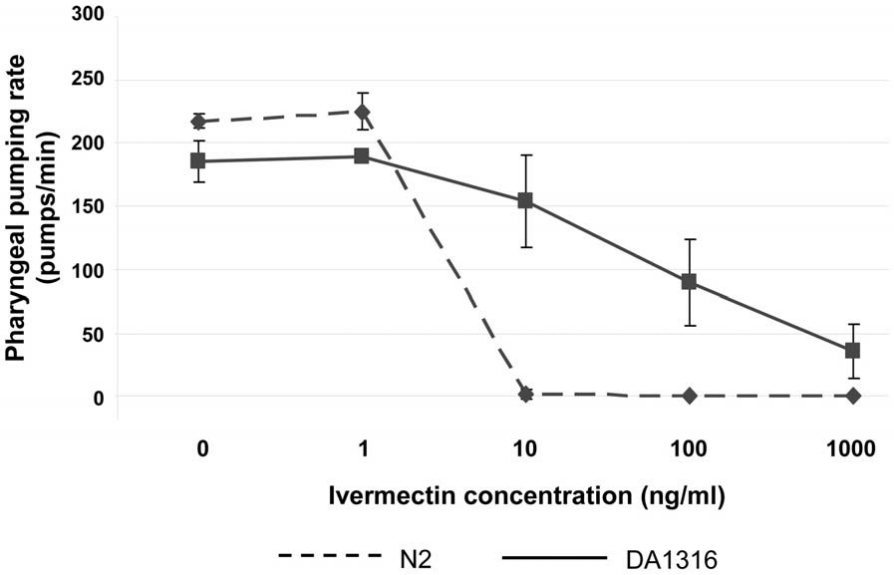
Pharyngeal pumping rate following 4 hr exposure of DA1316 and N2 to 0–1000 ng/ml (1.14 µM) IVM. Whilst strain DA1316 is more resistant to IVM induced pharyngeal paralysis, at concentrations greater than 100 ng/ml (0.1 µM) the pharyngeal pumping rate is significantly reduced. The pharyngeal pumping rates of five worms were counted at each concentration of IVM and the error bars represent the standard deviation.

Strain DA1316 is highly resistant to the effects of IVM on motility. Although wild-type worms (N2) exposed to 100 ng/ml (0.1 µM) IVM for as little as one hour exhibited a dramatic paralysis phenotype, exposure of strain DA1316 to the same concentration of drug for up to 6 hr had no visible effect on motility, as has been previously described (data not shown). In contrast, although the strain is resistant to the effects of IVM on the pharynx relative to wild type worms, we found that four hours exposure of DA1316 to IVM results in a significant decrease in pharyngeal activity (**Figure 1**). The number of pharyngeal pumps per minute is reduced by 50% following exposure to 100 ng/ml (0.1 µM) IVM. The drug is thought to elicit its effect on the pharynx via the AVR-15 subunit in pharyngeal GluCls [9], [12]. However, *avr-15* (*vu227*), presumed to be a null mutation of *avr-15*, does not appear to confer complete resistance to the effect of IVM on the *C. elegans* pharynx.

### Acute exposure of DA1316 to 0.1 µM and 1.1 µM IVM results in differential expression of a distinct set of genes that may be involved in the response to food deprivation

**Figure 2:**
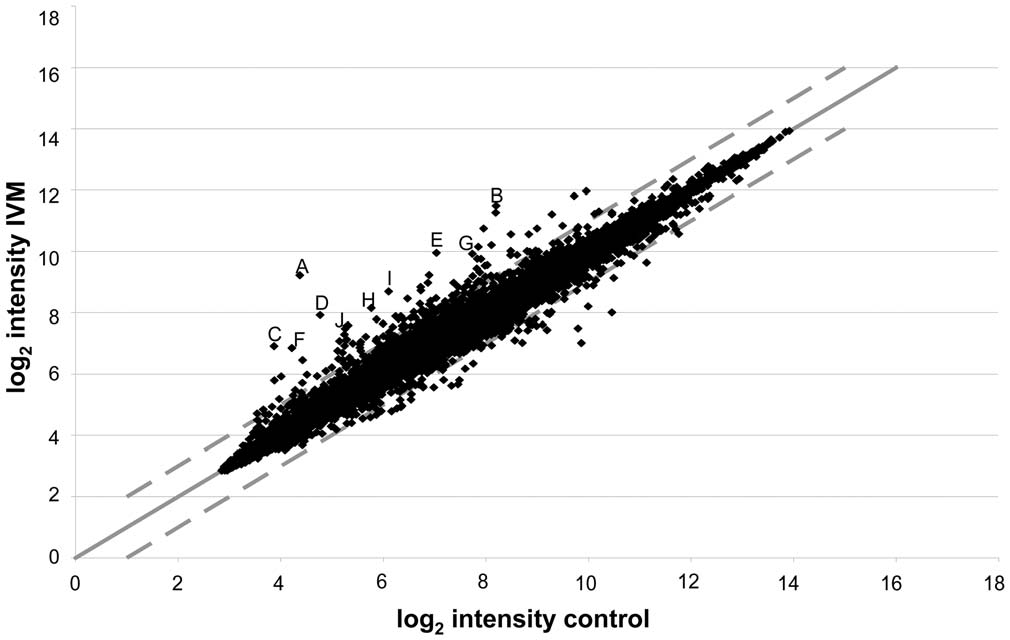
Model fitted log_2_ control chip intensity vs. log_2_ IVM (1 µg/ml, 1.1 µM) chip intensity. The scatter plot represents the entire 22625 probesets represented on the Affymetrix chips. The upper and lower dashed lines represent up-regulation greater than 2-fold and down-regulation greater than 2-fold respectively. The data points marked A-H represent the top 10 up-regulated genes in Table 2.

Microarray analysis was performed on RNA from five biological replicates of strain DA1316 exposed to 100 ng/ml (0.1 µM) IVM. Twelve probesets were considered to be significantly up-regulated and three considered to be significantly down-regulated (FDR<5%). The top 10 up-regulated genes, based on log_2_ fold change, are listed in **Table 1**. Many of the up-regulated genes are uncharacterised, but those with known or putative functions may have roles in fat metabolism. Given the low number of genes showing significant changes in expression the experiment was repeated using 1 µg/ml (1.1 µM) IVM in a similar manner. Again five drug-exposed and five matched controls underwent analysis. The rank products algorithm revealed 369 probesets to be significantly altered in expression with a FDR correction to 5% (216 up-regulated and 153 down-regulated). **Figure 2** summarises the microarray data and **Table 2** lists the top 10 up-regulated and down-regulated probesets based on log_2_ fold change. Full microarray data is available in **Table S2** and online at the GEO website, accession number GSE22660. Many genes are represented in the top up-regulated and down regulated lists for both the 0.1 µM and 1.1 µM experiments, including the presence of *mtl-1*, *scl-2* and *cyp-37B1* in the top four up-regulated genes, which suggests there is a consistent response at the two doses of drug.

**Table 1:** Top 10 up-regulated probesets based on fold change following 4 hr exposure of DA1316 to 100 ng/ml (0.1 µM) IVM.

Probeset	Gene ID	Log_2_ FC	FDR	*Ontology*
172744_at	*mtl-1*	1.59	0	*metallothionein*
184913_s_at	T22F3.11	1.44	0	*permease of major facilitator family KOG*
192737_at	*scl-2*	1.31	0	*sterol carrier-like protein*
189221_at	*cyp-37B1*	1.27	0	*cytochrome P450 (CYP4/19/26 subfamilies)*
186971_at	C23G10.11	1.23	0	*uncharacterised*
173729_at	T22F3.11	1.21	0	*permease of major facilitator family KOG*
183381_at	C50F7.5	1.12	1.00E-02	*uncharacterised*
186521_at	F21C10.10	1.10	1.11E-02	*uncharacterised*
173550_at	F45D3.4	1.08	1.25E-02	*uncharacterised*
190978_at	*sodh-1*	1.07	1.82E-02	*alcohol dehydrogenase class V KOG*

**Table 2:** Top 10 up-regulated and down-regulated genes based on fold change following 4 hr exposure of DA1316 to 1 µg/ml (1.1 µM) IVM.

*UP-REGULATED GENES*				
Probeset	Gene ID	Log_2_ FC	FDR	*Ontology*
172744_at	*mtl-1*	4.99	0	*metallothionein*
192737_at	*scl-2*	3.27	0	*sterol carrier-like protein*
186971_at	C23G10.11	3.20	0	*uncharacterised*
189221_at	*cyp-37B1*	3.09	0	*cytochrome P450 (CYP4/19/26 subfamilies)*
177613_at	F57G8.7	3.01	0	*uncharacterised*
177671_at	K03D3.2	2.83	0	*uncharacterised*
178900_s_at	F45D3.4	2.77	0	*uncharacterised*
187964_at	F54F3.3	2.51	0	*triglyceride lipase-cholesterol esterase KOG*
180946_at	*ilys-3*	2.51	0	*invertebrate lysozyme*
173335_s_at	*dod-3*	2.33	0	*down stream of daf-16*

*DOWN-REGULATED GENES*				
176939_at	*spp-23*	−2.79	0	*saposin-like protein family*
190404_s_at	*folt-2*	−2.55	0	*putative folate transporter*
179187_s_at	F46F2.3	−2.36	0	*uncharacterised*
189345_at	*pho-13*	−1.88	0	*predicted intestinal acid phosphatase*
192528_at	C35A5.3	−1.83	0	*uncharacterised*
187085_s_at	*gst-10*	−1.77	0	*glutathione-s-transferase*
190744_at	*ugt-63*	−1.77	0	*UDP-glucuronosyl/glucosyl transferase KOG*
175489_at	F18E3.11	−1.72	0	*uncharacterised*
177747_at	F58G6.9| *srm-3*	−1.72	0	*uncharacterised*
188441_at	F21F8.4	−1.70	0	*KOG- aspartyl protease*

Genes that showed significant changes in expression level following exposure of strain DA1316 to 1 µg/ml (1.1 µM) IVM (FDR<10%) were subject to ontology analysis. A less stringent FDR cut-off was used to widen the scope of the analysis and included 254 up-regulated and 186 down-regulated genes. The gene ontology terms associated with a minimum of two genes and with an associated EASE score (p-value) of ≤0.1, for both data sets, are described (**Table S3 and S4**). Only 72 up-regulated and 94 down-regulated genes were associated with significantly enriched ontology terms. This is likely due to the large number of completely uncharacterised genes in both sub-categories.

Up-regulated terms include *oxidoreductase activity*, *generation of precursor metabolites and energy*; *metabolic process*; *organic acid metabolic process*; *carboxylic acid metabolic process* and *catabolic process*. These terms are associated with several genes which may be important in fatty acid synthesis, breakdown and metabolism including five cytochrome P450 genes, two flavin containing monooxygenases (FMO) and three catalase genes; two short chain dehydrogenase genes and an alcohol dehydrogenase gene; a fatty acid desaturase gene and a gamma butyrobetaine hydroxylase (potentially involved in carnitine biosynthesis). The most significantly enriched biological process ontology term is *aging*, which includes *mtl-1*, *sodh-1*, *cyp-34A9* and *dod-3*. In addition, this group contains other down-stream targets of DAF-16, the sole *C. elegans* forkhead Box O transcription factor homologue and a mediator of insulin signalling [21], [34]. These include catalase genes (*ctl-2*, *ctl-1*); a gut esterase (*ges-1*); a fatty acid CoA synthetase gene (*acs-17*); a predicted isocitrate lyase/malate synthase (*gei-7*); and an acylsphingosine amidohydrolase (*asah-1*). All of these genes may be involved in fatty acid metabolism pathways. Furthermore, the only KEGG pathway term to be significantly enriched in the up-regulated gene list was f*atty acid metabolism*. This was associated with five genes: F54F3.4, *acs-2*, *sodh-1*, *acs-17* and F58F9.7.

The down-regulated gene-list is significantly enriched for the biological process term *carboxylic acid metabolic process*, which is associated with the fatty acid desaturase genes *fat-5*, *fat-6* and *fat-7*; and several hypothetical proteins with acyl-CoA thioesterase, acyl-CoA dehydrogenase, acyl-CoA oxidase, glycine dehydrogenase KOGs. Additionally, the fatty acid elongase genes *elo-2*, *elo-5* and *elo*-6; and genes involved in *lipid transport*, including the vitellogenins *vit-1*, *vit-3* and *vit-4*, are down-regulated. *Carbohydrate metabolic processes*, exemplified by the UDP-glucuronosyl transferases *ugt-12, ugt-46*; the lysozyme genes *lys-5* and *lys-6*; *gale-1* (a putative UDP-galactose-4-epimerase) and *ger-1* (a putative GDP-keto-6-deoxymannose 3,5-epimerase/4-reductase) are also enriched in the down-regulated gene list. Further evidence that IVM exposure does not result in a classical xenobiotic response is seen by the significant down-regulation of the molecular function terms *catalytic activity*, *oxidoreductase* and *transferase activity*. These terms are associated with six UDP-glucuronosyl/glucosyl transferases, four glutathione-s-transferases, one cytochrome P450 and one short-chain dehydrogenase; all of which represent gene families that would be expected to be up-regulated in a xenobiotic detoxification response.

**Figure 3:**
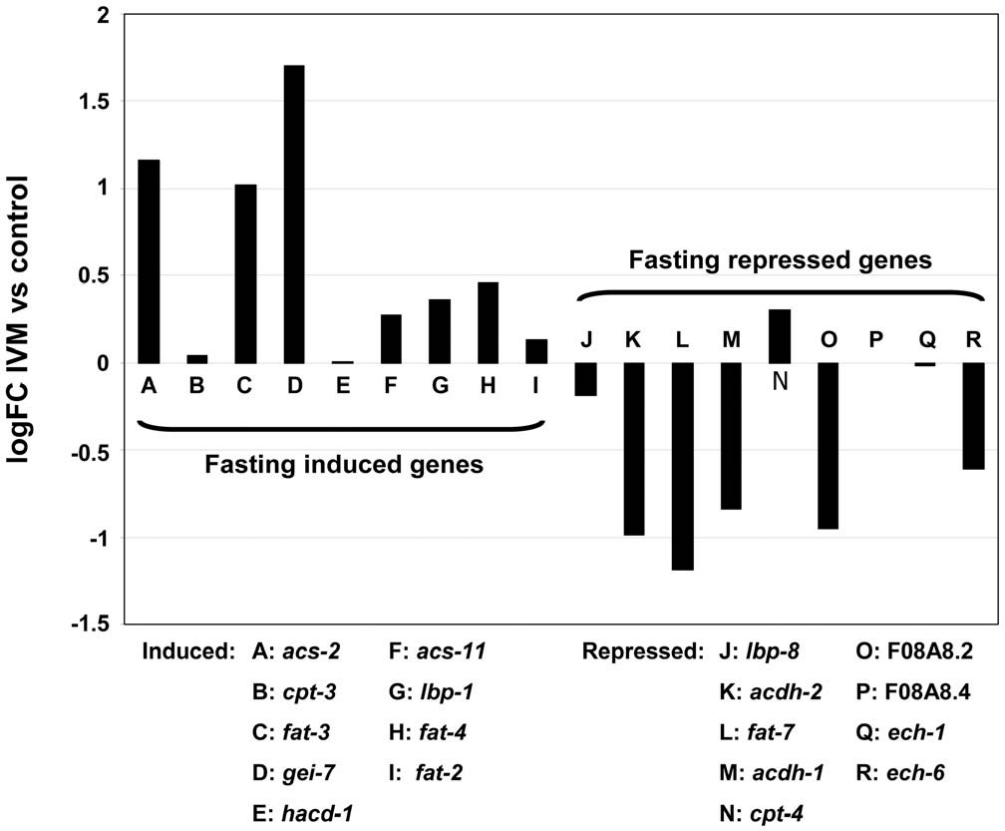
Differential expression of 18 fasting response genes*, in the 1 µg/ml (1.1 µM) IVM exposure microarray experiment. In general genes that were shown to be induced by fasting were also induced following exposure to IVM and fasting repressed genes were also repressed by IVM exposure. *Taken from van Gilst *et al.* 2005 [24].

The transcriptomic response to IVM exposure is inconsistent with a general stress response: A panel of genes that have been associated with stress, such as *hsp-70*, *gst-1*, *gst-38*, *sip-1* and *HSF-1*, show no significant change in expression in the microarray experiments; and others such as *hsp-16.1*, *hsp-16.49* and *gst-4* are in fact significantly down-regulated in the current study (**Table S5**). Instead, the overall analysis suggests that the predominant response is associated with an increase in lipid catabolism. We hypothesised that this was likely to be a result of short-term food deprivation associated with the reduced pharyngeal pumping, and hence feeding, which occurs following exposure of the DA1316 strain to IVM. In order to investigate this hypothesis further we compared our data to that of Van Gilst (2005) who identified 18 genes whose expression was significantly influenced by food withdrawal, using a quantitative real-time PCR screen of 97 candidate genes (**Figure 3**) [24]. The majority of the 18 genes identified by Van Gilst *et al.* as fasting response genes were similarly differentially expressed in microarray analysis of DA1316 exposed to IVM. This supports the hypothesis that the predominant response to IVM was secondary to its effect on the nematode pharynx. *cpt-4* was the only gene whose regulation was discordant, being up-regulated in response to IVM exposure and down- regulated in response to fasting. However, this gene is thought to encode a carnitine palmitoyl transferase, which would be expected to be up-regulated with increased fat catabolism.

### Real-time QPCR confirmation of microarray data

**Figure 4:**
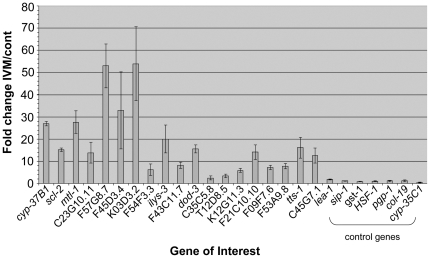
RTPCR assessment of gene expression following 4 hr exposure to IVM. Strain DA1316 was exposed to 1 µg/ml [1.1 µM] IVM (Virbamec) for 4 hr. Results are expressed as fold change relative to an unexposed control. Biological replicates were carried out in triplicate and the error bars represent the standard error. All genes proposed to be up-regulated by microarray were confirmed by RT-QPCR.

Genes which showed the greatest fold up-regulation following IVM exposure in the microarray experiments were further assessed using real- time QPCR. All of the genes examined that were proposed to be up-regulated by the microarray experiments were confirmed to be up-regulated by this technique (**Figure 4**). *sip-1*, *gst-1* and *HSF-1* are genes that have been associated general stress responses. These were included in the RT-QPCR analysis as negative controls and none showed any evidence of induction, supporting the earlier conclusion of a lack of a generalised stress response to IVM exposure. Expression of *cyp-35C1*, which we have shown to be up-regulated in response to albendazole exposure, was unchanged following IVM exposure [27]. In addition, *pgp-1*, an example of the p-glycoproteins that have been advocated to be responsive to chronic IVM exposure, showed no change in expression [35].

### *cyp-37B1*, *mtl-1* and *scl-2* represent novel genes involved in the response to food deprivation

*cyp-37B1*, *mtl-1* and *scl-2* were in the top four induced genes for both the 100 ng/ml (0.1 µM) and 1 µg/ml (1.1 µM) microarray experiments and their high level of induction was confirmed by RT-PCR. Additionally, both *cyp-37B1* and *mtl-1* have previously been shown to be responsive to exposure to other xenobiotics including clofibrate, β-naphthoflavone, PCB52, fluoranthene, progesterone and oestrogen [36]–[38]. Therefore, these genes were examined in more detail to characterize their response to both IVM exposure and food withdrawal.

**Figure 5:**
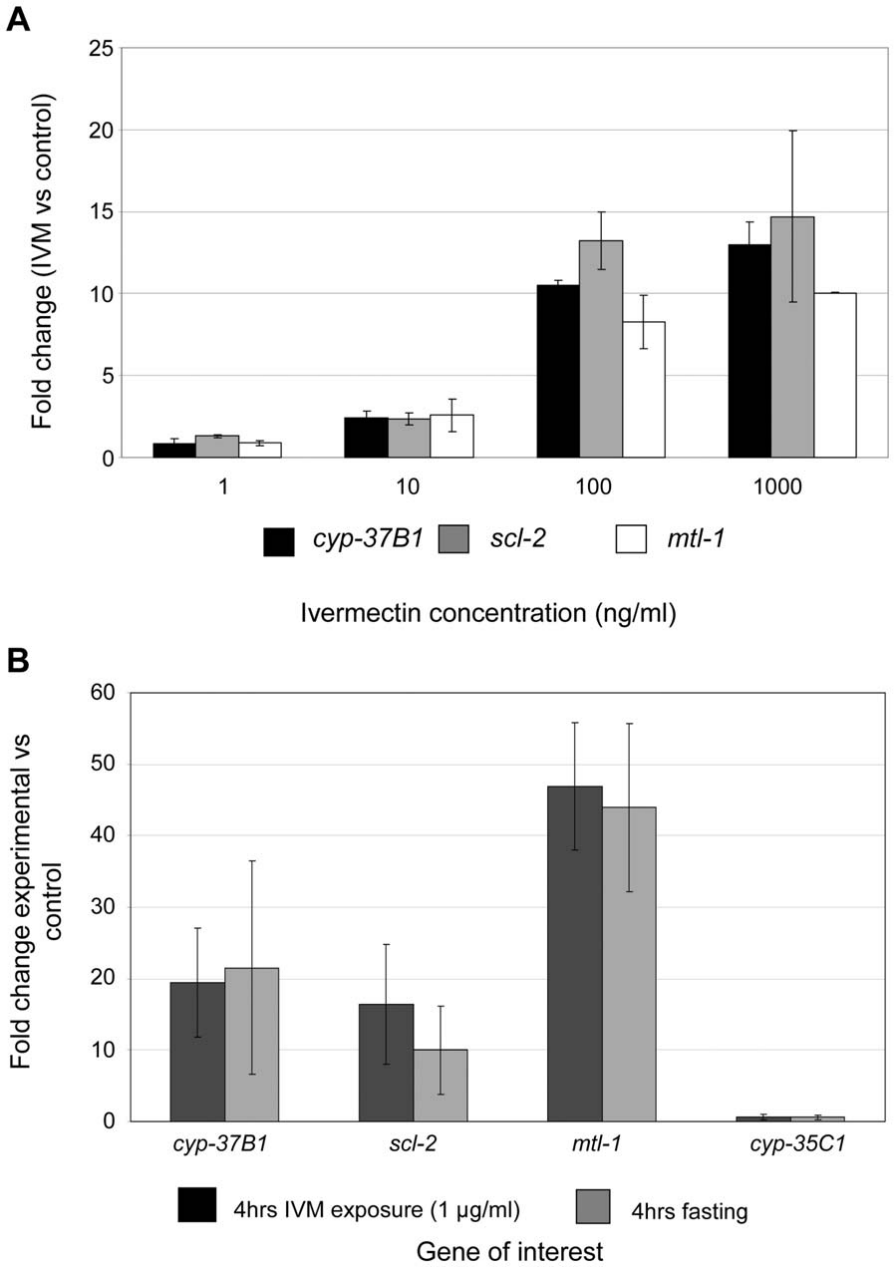
Up-regulation of *cyp-37B1*, *mtl-1* and *scl-2* in response to varying concentrations of IVM and fasting. **A.** Up-regulation of the genes of interest appears to occur in a dose-dependent manner. Biological replicates were carried out in duplicate and the error bars represent the standard error. **B.** There are no significant differences in the fold up-regulation of the genes investigated following exposure to 1 µg/ml (1.1 µM) IVM and 4 hr fasting. *cyp-35C1* (a gene transcriptionally induced by benzimidazole drug exposure [18]), included as a control, was unaffected by either treatment.

First, the dose responsiveness of *cyp-37B1*, *scl-2* and *mtl-1* was examined to relate the effects to those seen on the pharyngeal paralysis phenotype (**Figure 5A**). All respond to IVM in a dose responsive manner, with significant changes occurring in gene expression at doses of IVM greater than 10 ng/ml (11.4 nM). This is consistent with the drug concentration at which IVM begins to affect the pharynx of DA1316 (**Figure 1**). The induction of expression of these three genes following nutritional deprivation (food removal) for 4 hr was examined and all were found to be up-regulated (**Figure 5B**). Furthermore, the level of induction was very similar to that produced by a 4 hr exposure to 1 µg/ml (1.1 µM) of IVM, a concentration that results in almost complete pharyngeal paralysis in DA1316. *cyp-35C1*, included as a negative control, shows no change in either of the conditions examined in the current study.

### IVM responsive genes are predominantly expressed in the intestine and hypodermis

**Figure 6:**
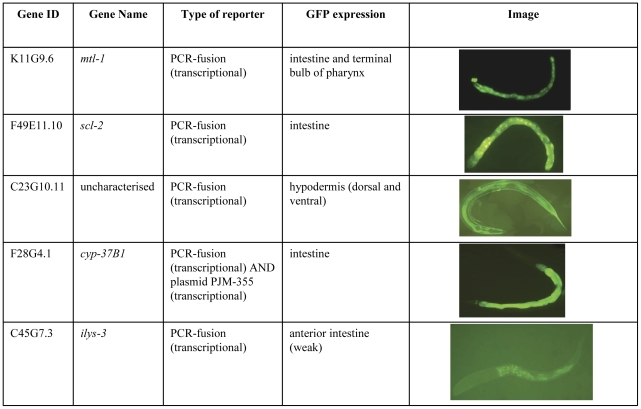
GFP expression patterns of IVM responsive genes. *mtl-1*, *scl-2*, *cyp-37B1* and *ilys-3* are expressed in the intestine and C23G10.11 is expressed in the hypodermal cells. These are both proposed sites of fat storage.

The expression patterns of a number of the induced genes were investigated. GFP transgenes of *mtl-1*, *cyp-37B1* and *scl-2* and *ilys-3* showed GFP expression in the intestinal cells (**Figure 6**), which is an important site for fat storage [39]. Additionally, *mtl-1* showed expression in the terminal bulb of the pharynx (**Figure 6**), as has been reported previously [39], [41]. In addition to the intestinal cells, the transcriptional reporter for *cyp-37B1* showed expression in two cells in the tail region, which were assumed to be the phasmid neurons. These are proposed to be chemosensory neurons involved in avoidance of noxious chemical stimuli [42]. C23G10.11 was expressed in the hypodermal cells, which are also proposed to be a site of fat storage [43].

## Discussion

### The transcriptional response of *C. elegans* strain DA1316 exposed to IVM is dominated by genes associated with fat mobilization and fatty acid metabolism

The transcriptional response of the *C. elegans* strain DA1316 (*avr-14(ad1302*); *avr-15(vu227)*; *glc-1(pk54)*) to IVM appears to be dominated by genes associated with the consequences of food deprivation, such as changes to fatty acid metabolism, many of which are conserved in higher mammals. Comparative analysis of our results with those of Van Gilst *et al.* (2005), provide compelling evidence for this (**Figure 3**) [24]. Furthermore we have directly demonstrated that the three genes most consistently induced by IVM exposure in our experiments (*mtl-1*, *cyp-37B1* and *scl-2*) are in fact directly inducible by food withdrawal in the absence of the drug. This conclusion is supported by the phenotype of the DA1316 strain when exposed to IVM. Although this strain, which has null mutations in three glutamate-gate chloride channels (*avr-14, avr-15* and *glc-1*), is highly resistant to the paralytic effects of the drug on body wall muscle, we found it was still sensitive (albeit less than wild type) to IVM induced pharyngeal paralysis (**Figure 1**). At doses of IVM of 100 ng/ml (0.1 µM) reduced pharyngeal pumping was evident with almost complete paralysis at 1 µg/ml (1.1 µM) (**Figure 1**). Hence the major effect of IVM on the DA1316 worms is likely to be a reduction in food intake. It should be noted that, in contrast, Ardelli *et al.* (2009) reported that an *avr-14*, *avr-15* and *glc-1* triple mutant showed no reduction in pharyngeal activity following 2.5 hr exposure to IVM at concentrations of up to 5 µM [44]. It may be that the longer period of exposure, 4 hr in the current study compared to 2.5 hr in that of Ardelli *et al.*, explains these differences.

We did not specifically investigate the effects of IVM-induced reduction of pharyngeal pumping rate on worm development and growth. However, it is interesting to note that DA1316 larvae grown on NGM containing IVM took longer to reach adulthood compared with those grown on drug-free plates; even at IVM doses as low as 11.4 nM (data not shown). At present, the extent to which the transcriptional response described here leads to metabolic changes that functionally compensate for reduced feed intake is not clear, and this will be an interesting area of future investigation.

These results also provide an interesting example of the care that needs to be taken when interpreting the transcriptional responses to drug exposure. At first sight some of the induced genes could be interpreted as being part of a specific xenobiotic response to IVM exposure, and perhaps represent candidate enzymes that may metabolize the drug. The cytochrome P450 gene *cyp-37B1* might be taken to be such a candidate. However, cytochromes P450 have roles in many constitutive biological processes in addition to drug metabolism, and in this case our results are more likely to implicate this enzyme in endogenous fatty acid metabolism rather than xenobiotic metabolism.

### Identification of novel genes induced in an early response to food deprivation

*C. elegans* has been established in the literature as a model for the investigation of fat metabolism and obesity [20], [21]. Whilst there is a huge amount of information regarding the medium and long term effects of food withdrawal on *C. elegans* and the induction of the dauer stage, little is known about the response of the worm to the earliest effects of food withdrawal. The identification of genes that are induced by short-term food deprivation was an unanticipated result of this study that is nevertheless potentially important. We have confirmed three of the genes (*mtl-1*, *cyp-37B1* and *scl-2*) induced by IVM exposure are truly induced by food withdrawal. Many of the other induced genes, most of which have not been ascribed a functional annotation, are likely to also be novel early fasting response/fat metabolism genes. An investigation of this and their role in these processes will be an interesting area of future study and may be relevant to fat mobilization and the molecular response to food deprivation in mammals.

*mtl-1*, *cyp-37B1* and *scl-2* have not been previously implicated in fasting responses or fat metabolism in *C. elegans* and so it is interesting to speculate on their roles in these processes. *mtl-1* is a metallothionein gene, which is inducible in response to heavy metal intoxication and stress adaptation [40]. Both rat and mouse *mtl* genes have been shown to be induced following fasting and may act as an antioxidant in the mouse [45]–[47]. However, metallothioneins have also been proposed to be involved in zinc signalling pathways within mammalian cells [48]. The *C. elegans mtl-1* gene is significantly divergent from the mammalian genes. In fact, *Ce-mtl-1* is the largest known metallothionein gene thus far investigated and therefore the function of MTL-1 may also be divergent in this species [40]. Interestingly, *mtl-1* has been noted to be up-regulated in response to several xenobiotics including progesterone, clofibrate and β-naphthoflavone and was also up-regulated in nematodes grown in axenic culture [36], [38], [49]. The phenotype of worms exposed to these xenobiotics was not reported in the literature. However, it seems likely that induction of *mtl-1* occurs under many different circumstances and may represent part of a common signalling pathway rather than an effector protein in the response to xenobiotic intoxication or food withdrawal.

The *mtl-1* transcriptional GFP reporter construct showed constitutive expression in the intestine. Previous reported studies have suggested that whilst *mtl-1* can be induced in the gut, constitutive expression is only found in the posterior bulb of the pharynx [41]. This would also suggest that *mtl-1* expression is higher in strain DA1316 than wild-type worms. The reason for this may be due to a level of pharyngeal dysfunction noted in glutamate-gated chloride channel subunit mutants, also noted in the slightly lower resting pharyngeal pumping rate of this strain (**Figure 1**) [10]. Hypothetically, this could result in slightly decreased food intake and a chronic up-regulation of the pathways involved in this response.

There have been no citations for *scl-2* in the literature and its function remains largely unknown. However, the gene encodes a sterol carrier-like protein domain and may potentially be involved in the transport of steroid hormones or lipid breakdown products. Up-regulation of a gene involved in such processes during food deprivation would be expected. Expression of *scl-2* appears to be confined to the intestinal cells at all stages. The intestinal cells represent a major site of fat storage in the nematodes [39]. Therefore, the localised expression of *scl-2* in the intestine makes it ideally placed for involvement in fat mobilization.

*cyp-37B1* represents a cytochrome P450 gene which encodes a CYP4/CYP19/CYP26 domain. Again, this gene has been shown to be up-regulated in response to other xenobiotics, but the phenotype of the exposed worms was not reported [36]–[38]. BLASTp analysis reveals that isoform 1 of CYP4V2 is a homologue of *C. elegans* CYP37B1 in the *Homo sapiens* genome (BLAST E-value 7.9×10^−98^, 90.6% length). Mutations of the gene encoding CYP4V2 have been associated with Bietti Crystalline Corneoretinal Dystrophy and the protein has recently been characterised as a fatty acid {omega}-hydroxylase [50], [51]. *cyp-37B1(RNAi)* suggests that this gene may have limited hydroxylase activity against eicosapentaenoic acid in *C. elegans* [52]. Therefore, it is possible that this cytochrome P450 is also involved in fat mobilisation in response to food deprivation. The expression of this gene in the intestine of the nematodes corroborates this hypothesis. However, there was also expression in the phasmid neurons, which may suggest involvement in chemosensation. Certainly, *C. elegans* appear to be able to sense IVM in their environment. Both wild-type (N2) and DA1316 animals will attempt to migrate off IVM containing NGM plates at concentrations of drug not high enough to cause immediate paralysis (data not shown).

Our initial hypothesis, that exposure of *C. elegans* to IVM would result in the up-regulation of xenobiotic metabolising enzymes, has been largely disproved in the current study. However, the pharyngeal dysfunction and associated decreased food intake caused by IVM, even in a highly resistant strain such as DA1316, has resulted in the identification of genes not previously implicated in food deprivation. Further investigation into the function of these genes should provide insight into the molecular pathways involved in reduced food intake, fasting and fat mobilization, both in nematodes and in higher species. Additionally, the recognition that IVM still induces a degree of pharyngeal paralysis in a mutant carrying null mutations in three glutamate-gated chloride channel subunits (avr-14, avr-15 and glc-1) suggests the existence of additional targets of the drug that are involved in nematode pharyngeal function.

## Supporting Information

Table S1 **Primer sets for real-time QPCR and GFP-fusion constructs.**
(XLS)

Table S2 **Complete list of genes with significant changes in expression following IVM exposure.** Log fold change in expression of all genes with a false discovery rate of less than 5% in both the 100 ng/ml (0.1 µM) and 1 µg/ml (1.1 µM) IVM experiments are listed.
(XLS)

Table S3 **Ontology terms associated with up-regulated genes in response to exposure of DA1316 to 1 µg/ml (1.1 µM) IVM for 4 hr.**
(XLSX)

Table S4 **Ontology terms associated with down-regulated genes in response to exposure of DA1316 to 1 µg/ml (1.1 µM) IVM for 4 hr.**
(XLSX)

Table S5 **Log fold change in expression of “classic” stress response genes following IVM exposure (1 µg/ml, 1.1 µM).**
(XLS)
