# Correction: The Transcriptional Response of *Caenorhabditis elegans* to Ivermectin Exposure Identifies Novel Genes Involved in the Response to Reduced Food Intake

**DOI:** 10.1371/annotation/259d774f-6c58-4df8-bce5-74a3c9160270

**Published:** 2012-08-10

**Authors:** Steven T. Laing, Al Ivens, Victoria Butler, Sai P. Ravikumar, Roz Laing, Debra J. Woods, John S. Gilleard

There was an error in the fourth paragraph of the "Identification of novel genes induced in an early response to food deprivation" section of the Discussion.

The text currently reads: "There have been no citations for scl-2 in the literature and its function remains largely unknown. However, the gene encodes a sterol carrier-like protein domain and may potentially be involved in the transport of steroid hormones or lipid breakdown products. Up-regulation of a gene involved in such processes during food deprivation would be expected. Expression of scl-2 appears to be confined to the intestinal cells at all stages. The intestinal cells represent a major site of fat storage in the nematodes [39]. Therefore, the localised expression of scl-2 in the intestine makes it ideally placed for involvement in fat mobilization."

The correct text is: "The function of scl-2 remains largely unknown, but the gene encodes a predicted extracellular protein belonging to the sperm coating protein (SCP)-like domain containing protein family. Expression of scl-2 appears to be confined to the intestinal cells at all stages, which represent a major site of fat storage in the nematodes [39]. Localised expression in the intestine makes it ideally placed for involvement in fat mobilization, but we cannot speculate on its precise role in the response to reduced food intake further."

The change does not impact the rest of the manuscript or change the findings therein.

